# Diurnal profiles of pedometer-determined physical activity in chronically ill and mobility-limited older adults: a cross-sectional study

**DOI:** 10.1186/1471-2458-14-1268

**Published:** 2014-12-13

**Authors:** Anna Mai, Alexander Bloch, Renate Klaaßen-Mielke, Petra Platen, Timo Hinrichs

**Affiliations:** Department of Medical Informatics, Biometry and Epidemiology, Ruhr-University Bochum, Springorumallee 5, 44795 Bochum, Germany; Department of Sports Medicine and Sports Nutrition, Ruhr-University Bochum, Gesundheitscampus Nord 10, 44801 Bochum, Germany; Swiss Paraplegic Research, Guido A. Zäch Strasse 4, 6207 Nottwil, Switzerland; Department of Health Sciences and Health Policy, University of Lucerne, Frohburgstrasse 3, P.O. Box 4466, 6002 Lucerne, Switzerland

**Keywords:** Aged, Community-dwelling, Objective measurement, Step count, Moderators

## Abstract

**Background:**

The aim of this study was to analyze diurnal profiles of physical activity for community-dwelling adults aged 70 years and over, and to explore the moderating effect of sex, age, morbidity, mobility limitation, and season on physical activity throughout the day.

**Methods:**

A sample of 149 primary health care patients (mean age 79.5 ± 5.2 years, 74.5% females) was included in the analyses. Participants’ physical activity was measured on up to six consecutive days via Omron Walking Style Pro HJ-720IT-E2 pedometer. Step count per day and per hour, and pedometer wear time were descriptively analyzed. A repeated measures ANOVA with physical activity per hour as dependent variable was performed to analyze the moderating effect of sex, age, morbidity, mobility limitation, and season on diurnal profiles of physical activity. The diurnal profile for the total sample and adjusted diurnal profiles for subgroups are presented.

**Results:**

Participants’ daily step count averaged 3280 ± 1873 steps/day. They wore the pedometer for 14.2 ± 1.7 hours per day and walked on average 234 ± 139 steps per hour. With respect to diurnal profiles, there were two peaks at 10–11 AM (mean [95%-confidence interval]: 382 [329–435] steps) and at 3–4 pm (313 [261–365] steps) interrupted by a period of lower activity with a low point at 1–2 pm (229 [190–268] steps). A mobility limitation, defined by use of a cane or a rollator, had a significant moderating effect (p = 0.0237) on diurnal physical activity.

**Conclusions:**

This is the first study to explore pedometer-determined diurnal profiles of physical activity in chronically ill and mobility-limited older adults. Prolonging periods of elevated physical activity in the afternoon while respecting individual daily routine and commitments could be one option for facilitating the integration of physical activity and for making it a habit in older adults’ daily lives. The use of a walking aid seems to be an indicator for a quite low activity plateau during the second half of the day. People who use walking aids should be motivated to increase their physical activity during afternoon; this might help to increase the overall low physical activity level of this vulnerable subgroup of older adults.

## Background

Regular physical activity has the potential to preserve or even improve health and health-related quality of life up until old age. Numerous studies have proven this impressively, even in chronically ill, functionally impaired, or very old adults [1]. However, for the most part, the activity behavior of the older population does not conform to current recommendations [2–5].

Furthermore, physical activity levels are even lower among persons affected by chronic diseases or functional limitations [6, 7]. In order to derive reasonable health promotion and intervention strategies, it is important to better understand the physical activity behavior of older adults, and particularly those being chronically ill and functionally impaired.

A wide range of epidemiological studies have examined physical activity in older adults [4, 8–10]. So far, studies either focused on total activity over a certain period of time, e.g., per week or per day, usually in order to evaluate older adults’ physical activity level with respect to current guidelines, or on correlates of overall physical activity [11, 12], or on motivators and barriers [13, 14]. Without any doubt, these studies provide fundamental information [10]. There is, however, scant knowledge about the timing of physical activity during the course of a day.

One of the few studies to report on daily physical activity profiles was a one-case study that explored the physical activity behavior of an older woman living alone [15]. This study assessed physical activity by means of a complex monitoring system installed on a computer in a private household. Since the hourly activity recording was limited to physical activities at home, and due to the one-case study character, it is nearly impossible to generalize the results with regard to older adults’ physical activity behavior. Another study investigated diurnal profiles of physical activity by accelerometer in patients with chronic obstructive pulmonary disease (COPD) of all ages [16]. To the authors’ knowledge, no studies have been published to date that evaluate diurnal profiles of physical activity with a focus on community-dwelling older adults with chronic diseases and functional limitations. An explorative analysis of physical activity in the course of the day may provide new and interesting insights regarding the physical activity behavior of those older adults, and might help to generate hypotheses on optimized physical activity promotion.

Physical activity behavior is considered to be influenced by a complex interaction of sociodemographic, physical, psychological, social, environmental and sociopolitical factors [12, 17]. Sex and age are two of the most consistent factors influencing physical activity [11, 12]. Physical health status and functional limitations have also been shown to be highly important determinants of physical activity behavior in older adults [7, 12, 13, 18]. Additionally, there is increasing evidence that physical activity behavior is subject to seasonal variations [7, 19]. Day length, maximum temperature and sunshine duration were shown to be associated with physical activity levels [20]. We hypothesize that sex, age, morbidity, mobility limitation, and season might not only influence overall physical activity, but may also differently impact older adults’ physical activity in the course of the day. To date, no data has been reported on this topic.

Consequently, the aim of the present study was to analyze diurnal profiles of physical activity for community-dwelling, chronically ill and mobility-limited adults aged 70 years and over, and to explore the moderating effect of sex, age, morbidity, mobility limitation, and season on physical activity in the course of the day.

## Methods

### Design and participants

The present study refers to cross-sectional data collected at baseline (before randomization) in the HOMEfit study [21], a randomized controlled trial exploring the effects of a home-based exercise program for chronically ill and mobility-limited, yet community-dwelling adults aged 70 years and over. The HOMEfit concept was to access these hard-to-reach subjects via their general practitioner (GP). Fifteen GP practices, belonging to a network of “research practices” administered by the Institute of General Practice and Family Medicine, University of Witten/Herdecke in Western Germany, participated in the study.

A multi-stage recruitment procedure was used to assess all GP patients aged 70 years and over who had seen their GP within the past 6 months (n = 5990 in 15 practices) for all predefined inclusion and exclusion criteria. A detailed analysis of the recruitment steps has already been published [22]. In brief, all patient records at a GPs practice were pre-screened for several eligibility criteria by a practice nurse and a study physician. Patients who seemed to be eligible based on their records (n = 1214) were invited for the full screening of inclusion and exclusion criteria at their GP’s practice. 434 patients responded to the invitation and took part in the face-to-face eligibility screening. 245 patients fulfilled all inclusion and no exclusion criteria (see below), and gave written informed consent to participate in the study. Please, see the recruitment paper for detailed information on recruitment success rates, or reasons for exclusion and non-participation [22].

The study was approved by the University of Witten/Herdecke ethics committee.

### Inclusion and exclusion criteria

Important inclusion criteria were 1) age ≥ 70 years, 2) community-dwelling (not institutionalized), 3) chronically ill (diagnosed with at least 1 of 11 specified chronic diseases [see Table 1]), 4) mobility-limited (i.e., having at least some self-reported problems walking 2 km or climbing a flight of stairs) but not in need of assistance to walk or not wheelchair-bound, 5) with only low or sedentary physical activity levels (exercise, sporting activities or leisure activities that cause sweating and/or harder breathing for < 2 hours per week, and walking outdoors for < 4 hours per week), and 6) no highly increased risk of medical adverse events. For detailed information on all inclusion/exclusion criteria, see the study protocol of the intervention study [21].

**Table 1:** **Participants’ characteristics**

	Total (n = 149)
**Socio-demographic factors (%)**	
Female	74.5
Living alone (n = 148)	58.1
SES* (n = 123)	
Low	39.0
Middle	51.2
High	9.8
**Anthropometric data (mean ± SD)**	
Age [years]	79.5 ± 5.2
BMI [kg/m^2^]	30.3 ± 5.5
**Functional tests (mean ± SD)**	
Timed up-and go [s]	12.8 ± 4.5
Grip strength (dominant hand) [kg]	24.2 ± 8.3
Chair-rise [s]	20.0 ± 10.2
Tandem stand [s]	8.7 ± 2.4
**Chronic diseases (%)**	
Hypertension	89.3
Type 2 diabetes	34.2
Chronic ischemic heart disease	28.9
Heart failure	34.9
Peripheral arterial disease	13.4
COPD	21.5
Chronic kidney disease	19.5
Spinal osteochondrosis	67.8
Osteoarthritis of the hip	47.7
Osteoarthritis of the knee	60.4
Osteoporosis	20.1
Number of specified diseases	
1	4.7
2-3	30.2
4-5	38.9
≥ 6	26.2
**Mobility limitation and falls (%)**	
No walking aid	49.7
Cane	28.2
Rollator	22.1
Falls in the last 12 months (n = 147)	
0	71.4
1-2	23.8
≥ 3	4.8

*SD* standard deviation; *COPD* chronic obstructive pulmonary disease;

*Socioeconomic status determined by education, job and income.

### Assessment of physical activity

Participants received a pedometer during the baseline assessment at the GP’s practice. They were instructed to wear the pedometer on six consecutive days following the assessment before randomization for the trial. The pedometer model used was a Walking Style Pro HJ-720IT-E2 (Omron Healthcare Co., Kyoto, Japan) with a piezoelectric sensor. The pedometer had to be worn on the waistband/belt perpendicular to the ground [23]. This was reported to be the most precise mounting position [24] out of four mounting positions proposed by the manufacturer for this model. The correct mounting on the waistband/belt was demonstrated by the assessors and practiced by the older adults to ensure correct application. Participants were instructed to wear the device immediately after getting up in the morning until they went to sleep, and to document whether they had worn the device for the whole day (yes/no). The device has a 41-day memory. The stored activity data was downloaded using the associated software, which generates an hourly step output.

### Assessment of patient characteristics

Trained assessors measured anthropometric parameters (height, weight) as well as physical function (mobility [timed up-and-go], functional leg strength [chair rise], hand grip strength [dynamometer], and balance [tandem stand]) during the baseline assessment at the GP’s practice. The GP documented chronic diseases (essential hypertension, type 2 diabetes, chronic ischemic heart disease, heart failure, peripheral arterial disease, COPD, chronic kidney disease, spinal osteochondrosis, osteoarthritis of the hip, osteoarthritis of the knee, and osteoporosis with or without pathological fracture). Year of birth and sex were documented during patient recruitment. Sociodemographic factors (including socioeconomic status and living situation) were assessed during computer-assisted telephone interviews in line with German epidemiological standards [25]. Current walking ability (no walking aid/cane/rollator) and frequency of falls (12-month recall) were also assessed by telephone interview (see also Hinrichs et al. [21]).

### Data analysis

The following criteria were defined in order to obtain valid and reliable data on usual daily physical activity: 1) the participants had to wear the pedometer all day. 2) They had to reach a minimum of 300 steps per day. Days with less than 300 steps were deleted. Each day with a minimum of 300 steps was considered for analysis. 3) Finally, participants had to wear the pedometer on at least 2 days between Monday and Saturday (M-S days) and on a Sunday. Past studies have reported that 3 days are adequate for measurement of older adults’ usual physical activity by pedometer, due to low day-to-day variability of physical activity in this age group [26]. However, older adults’ physical activity level has been shown to be lower on Sundays than on the other days of the week [27]. Our study therefore aimed to obtain physical activity data from at least 3 days, including a Sunday. No plausible suggestions were available in the literature for a lower cut-off for older adults’ daily activity. In a review on expected values for pedometer-determined physical activity in older populations Tudor-Locke et al. pointed out: “[…] Data for any single day indicating < 1,000 steps were removed […]. No other study described any other treatment for […] extreme values” [10]. A lower cut off point of 1000 steps, however, seemed too high and thus inappropriate for a chronically ill and mobility-limited population. Another review on expected step counts for older adults and special populations presented the step cadence of patients with peripheral arterial disease [28]. During a 30-minute walk under free-living conditions, they accumulated 35 steps/min. This would results in 300 steps after only 8.5 minutes of walking. The stride length of a sample of older adults recruited from nursing facilities in Germany was 41.1 ± 10.7 cm (own data, unpublished yet). 300 steps would correspond to approximately only 120 m walking distance on a whole day. Against this background, 300 steps were assumed to be a plausible minimum amount for a whole day measurement of physical activity in this specific study population.

Due to a varying number of M-S days with step data per participant (minimum 2, maximum 5 M-S days), physical activity per day was calculated as weighted mean for each participant ([6 × average of M-S steps + Sunday steps]/7) in order to obtain a value for physical activity per day representing his/her total week. Hourly pedometer output was analyzed the same for each participant, e.g., [6 × average of M-S steps between 6 am and 7 am + Sunday steps between 6 am and 7 am]/7. Pedometer wear time was counted in full hours beginning with the first steps in the morning until the last steps in the evening. Averaged steps per hour were calculated as steps per day divided by pedometer wear time for each participant.

Sample characteristics were analyzed descriptively for the total sample. Physical activity per day and per hour, and pedometer wear time were analyzed descriptively for all participants and by sex (women/men), age (< 80 years/≥ 80 years), morbidity (< 6 chronic diseases/≥ 6 chronic diseases), mobility limitation (no walking aid/cane or rollator), and season (autumn-winter/spring-summer). Season was defined by date of baseline assessment: autumn/winter = October-March, spring/summer = April-September, cut points corresponding well to changes of day length and day light hours.

A repeated measures ANOVA with physical activity per hour as dependent variable was performed to analyze the moderating effect of sex, age, morbidity, mobility limitation, and season on diurnal profiles of physical activity. The level of significance was set to p < 0.05. Due to inhomogeneity of variances Greenhouse-Geisser adjusted p-values were used.

Hourly step data were illustrated as diurnal profile of physical activity for the total sample (means and 95%-confidence intervals), and as adjusted diurnal profiles for each subgroup (estimated marginal means and 95%-confidence intervals). Physical activity between 0 and 6 am was recorded only in very few instances, graphs of diurnal profiles thus cover the period between 6 am and 12 pm.

## Results

### Participants’ characteristics

**Figure 1:**
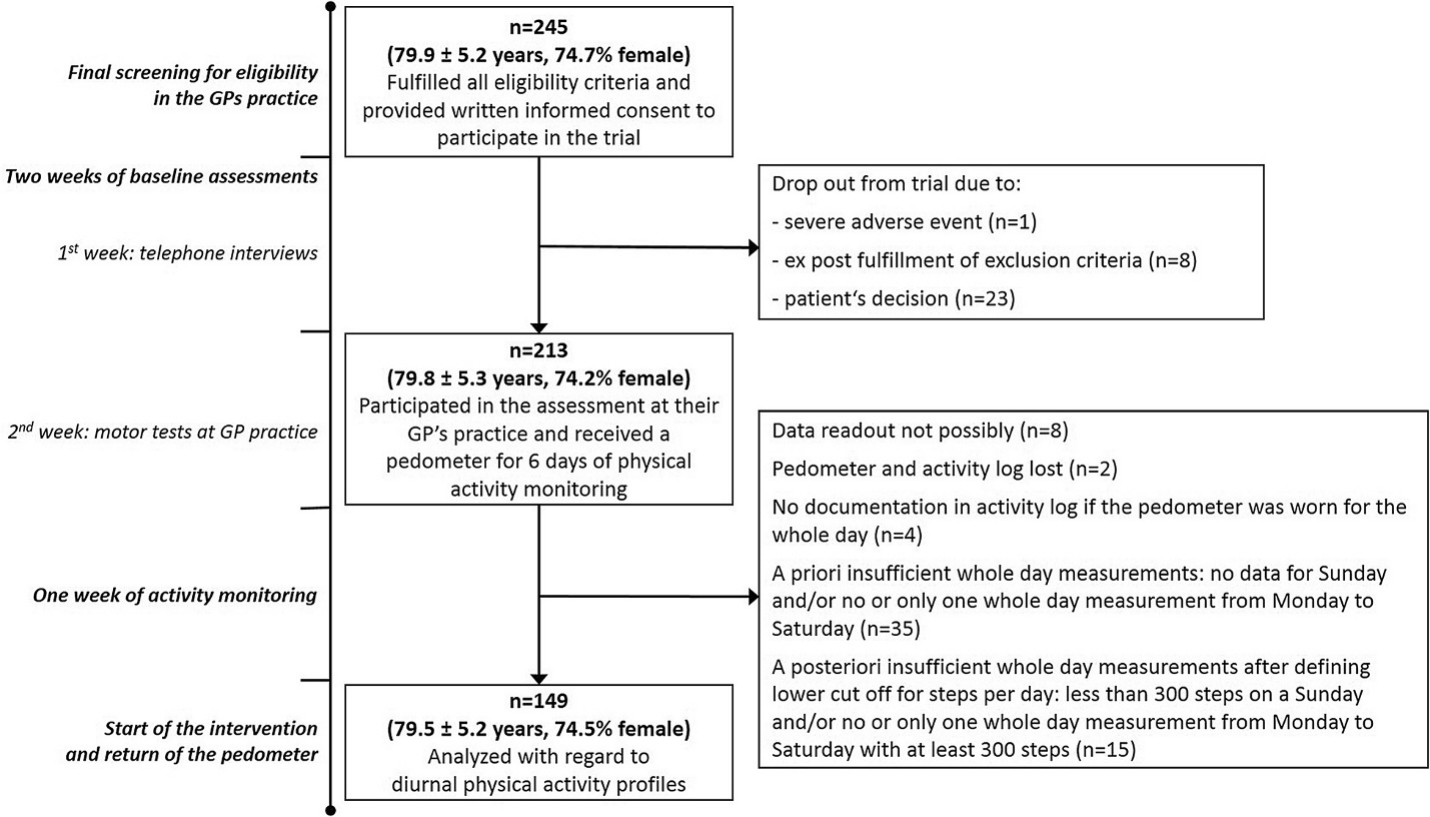
**Flow chart on patient selection from 245 patients providing written informed consent to 149 patients analyzed with regard to diurnal physical activity profiles.**

Out of the 245 patients (79.9 ± 5.2 years, 74.7% female) providing written informed consent, 213 patients (79.8 ± 5.3 years, 74.2% female) took part in the baseline assessment at the GP’s practice including pedometer release. A total of 149 participants (mean age 79.5 ± 5.2 [70–94] years, 74.5% female) provided full activity data, met the defined analysis criteria, and was thus analyzed with regard to daily physical activity behavior. A flow chart on patient selection and reasons for exclusion is presented in Figure 1.

The characteristics of the 149 participants analyzed are presented in Table 1. 65.1% had four or more chronic illnesses, 50.3% used a walking aid (cane or rollator), and 28.6% had fallen at least once in the previous 12 months.

### Physical activity parameters

The median number of pedometer measurement days was six days. 7.4% of participants fulfilled the minimum criterion of a three day measurement including Sunday. Pedometer wear time, steps per day and per hour are presented in Table 2.

**Table 2:** **Pedometer-determined physical activity parameters**

	Steps per day	Pedometer wear time per day	Steps per hour
	Mean ± SD	Mean ± SD	Mean ± SD
**All** (n = 149)	3280 ± 1873	14.2 ± 1.7	233 ± 138
**Sex**			
Women (n = 111)	3350 ± 1931	14.2 ± 1.7	238 ± 142
Men (n = 38)	3075 ± 1700	14.2 ± 1.7	218 ± 123
**Age group**			
< 80 years (n = 74)	3722 ± 1978	14.3 ± 1.7	264 ± 144
≥ 80 years (n = 75)	2844 ± 1663	14.1 ± 1.8	203 ± 124
**Morbidity**			
< 6 chronic diseases	3409 ± 1961	14.1 ± 1.7	243 ± 143
≥ 6 chronic diseases	2917 ± 1563	14.4 ± 1.7	205 ± 116
**Mobility limitation**			
No walking aid (n = 74)	3727 ± 1935	14.1 ± 1.8	267 ± 146
Cane or rollator (n = 75)	2839 ± 1710	14.2 ± 1.7	199 ± 121
**Season**			
Autumn/winter (n = 61)	3413 ± 2056	14.1 ± 1.8	245 ± 152
Spring/summer (n = 88)	3187 ± 1741	14.2 ± 1.7	225 ± 127

*SD* standard deviation.

Participants wore the pedometer for 14.2 ± 1.7 hours per day. Their physical activity averaged 3280 ± 1873 steps per day and 233 ± 138 steps per hour. While pedometer wear time did only marginally differ between subgroups, there were considerable descriptive differences in physical activity levels between younger and older participants (3722 ± 1978 steps per day vs. 2844 ± 1663 steps per day; 238 ± 142 steps per hour vs. 218 ± 123 steps per hour), and participants without and with a walking aid (3727 ± 1935 steps per day vs. 2839 ± 1710 steps per day; 267 ± 146 steps per hour vs. 199 ± 121 steps per hour); see Table 2.

### Diurnal profiles of physical activity

**Figure 2:**
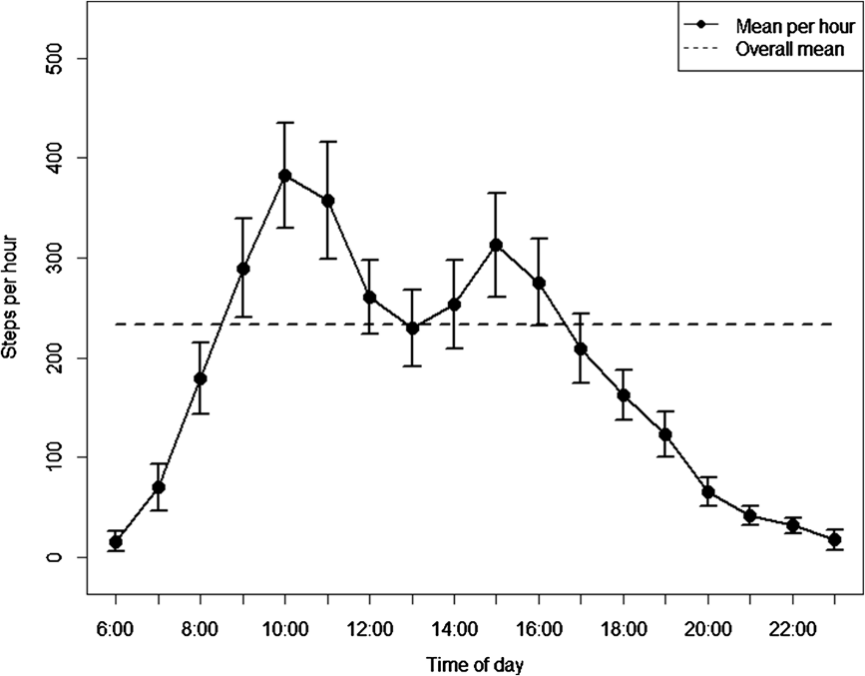
**Diurnal profile of physical activity for all subjects (means ± 95%-confidence intervals).**

The diurnal profile for the total sample shows 2 peaks and 1 low point of physical activity in the course of the day (Figure 2). From 6 am on, physical activity rose until a first peak between 10 and 11 am, with a mean step count of 382 [95%-confidence interval: 329–435] steps per hour. In the course of the early afternoon, physical activity decreased again and attained a low point between 1 and 2 pm (229 [190–268] steps per hour). The second peak of physical activity was reached between 3 and 4 pm (313 [261–365] steps per hour). From 4 pm on, physical activity declined rapidly until 9 pm with hardly any activity recorded between 9 pm and midnight.

**Figure 3:**
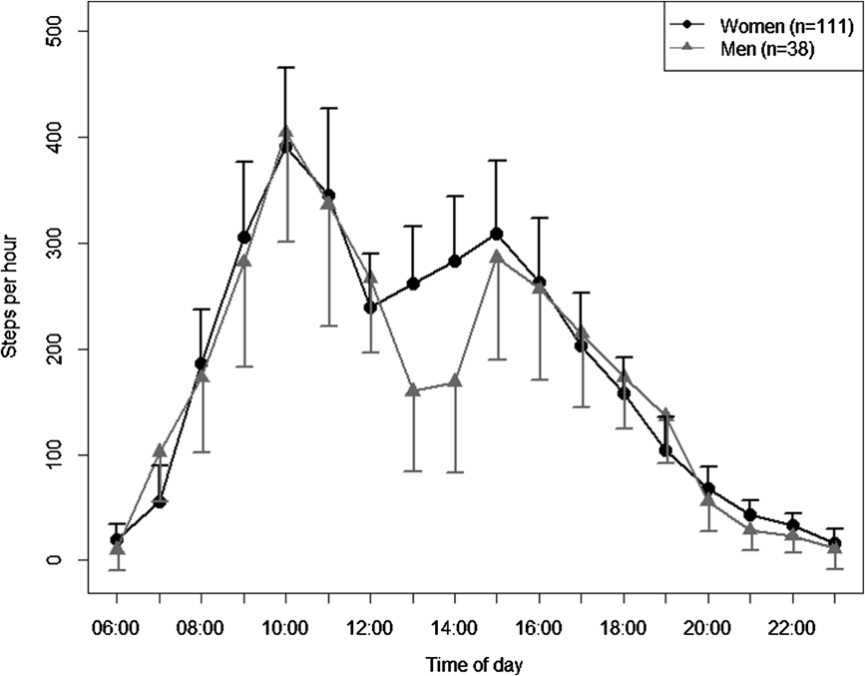
**Diurnal profiles of physical activity by sex adjusted for all other variables in the model (estimated marginal means ± 95%-confidence intervals).**

**Figure 4:**
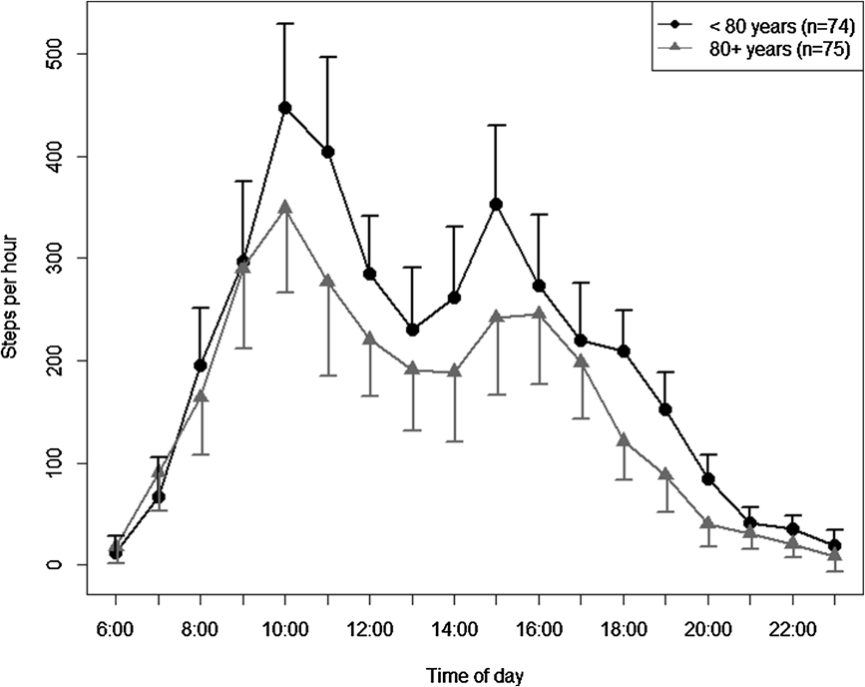
**Diurnal profiles of physical activity by age adjusted for all other variables in the model (estimated marginal means ± 95%-confidence intervals).**

**Figure 5:**
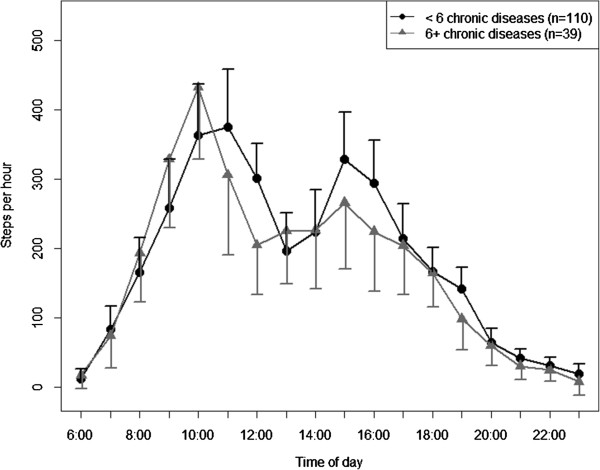
**Diurnal profiles of physical activity by morbidity adjusted for all other variables in the model (estimated marginal means ± 95%-confidence intervals).**

**Figure 6:**
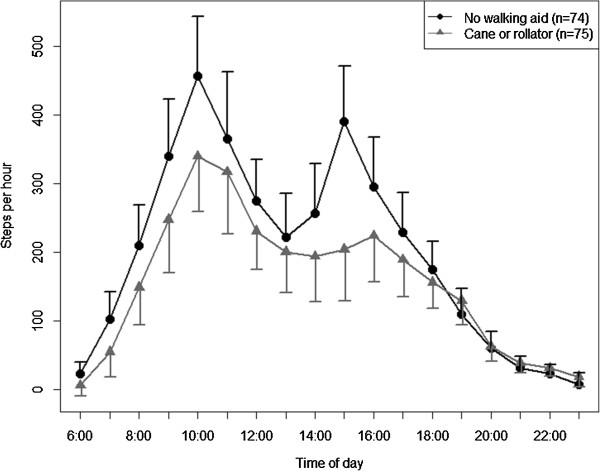
**Diurnal profiles of physical activity by mobility limitation adjusted for all other variables in the model (estimated marginal means ± 95%-confidence intervals).**

**Figure 7:**
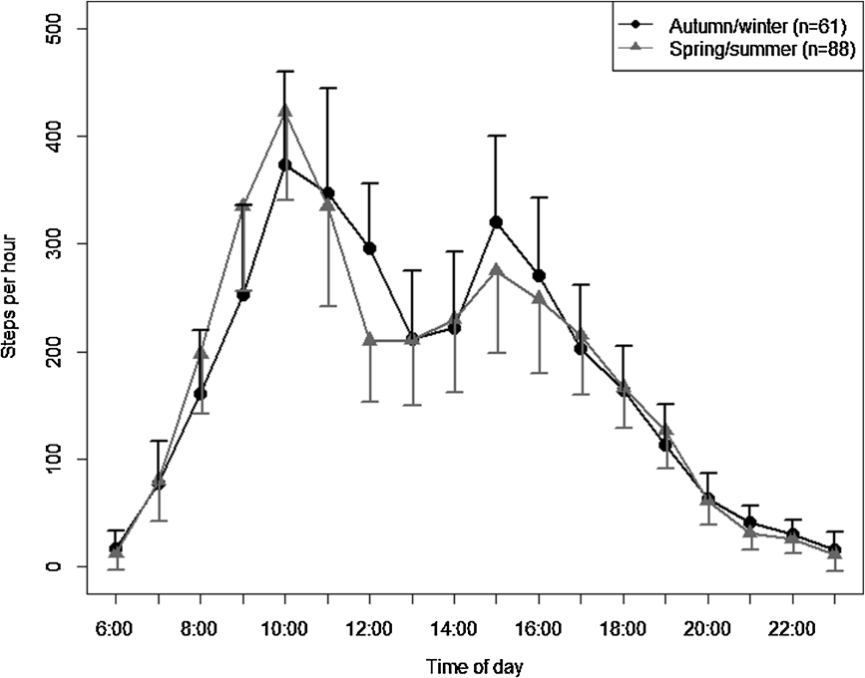
**Diurnal profiles of physical activity by season adjusted for all other variables in the model (estimated marginal means ± 95%-confidence intervals).**

The adjusted diurnal profiles for subgroups are presented in Figures 3, 4, 5, 6 and 7. In principal, the general trend for the total sample was noticeable, with slight variations, in the group-specific evaluations as well. The repeated measures ANOVA initially revealed significant differences in overall physical activity (between subject effects) between age groups (p = 0.0076) and mobility limitation groups (p = 0.0136), respectively adjusted for all the other variables in the model. The analysis of within subject effects revealed a significant moderating effect (p = 0.0237) for mobility limitation on physical activity throughout the day.

The diurnal profiles for participants without a walking aid and for those in need of a cane or a rollator are shown in Figure 6. The profiles are adjusted for sex, age, morbidity, and season. Comparable to the overall trend, the diurnal profiles exhibited a peak between 10 and 11 am (estimated marginal means [95-CI]; no walking aid: 457 [369;544] steps per hour vs. cane or rollator: 340 [258;421] steps per hour), and a low point between 1 and 2 pm (no walking aid: 221 [157;286] steps per hour vs. cane or rollator: 200 [141;260] steps per hour). Until this point in time the curves run more or less parallelly, though on a lower level for those using a walking aid. From then on, curves are markedly different in the course of the afternoon. While participants without a walking aid reached a second peak of 390 [309;471] steps per hour between 3 and 4 pm, participants using a walking aid had virtually no increase in physical activity any more: the lowest step count of 194 [127;261] steps per hour was measured between 2 and 3 pm, the highest activity was 224 [156;291] steps per hour between 4 and 5 pm. From then on, curves were more or less identical with a sharp decrease in physical activity until midnight.

## Discussion

This study evaluated pedometer-determined diurnal profiles of physical activity in community-dwelling, older primary health care patients and explored the moderating effect of sex, age, morbidity, mobility limitation, and season on physical activity behavior in the course of the day.

### Overall diurnal physical activity profile

Analysis of the overall diurnal profile of physical activity revealed two peaks, with one peak in the late morning, a second peak in the afternoon, and a low point in between. The low level of physical activity in the early afternoon could be explained by an afternoon nap. Studies showed that older people usually take a nap after lunch or in the early afternoon [29]. One study elicited data on the sleeping habits of 1497 adults aged 55–85 years and over by telephone interview [30]. The results revealed the frequency of individuals taking afternoon naps increased with age. For 25% of the 288 participants aged 75–85 years, an afternoon nap was an integral part of their daily routine. Chronically ill and mobility-limited older people were shown to have even higher volumes of afternoon naps [31]. With respect to afternoon napping habits between men and women, a study with 10126 participants revealed that men took afternoon naps more often and longer than women [31]. This finding might explain men’s lower physical activity level in general and during the early afternoon compared to women in the present study, though sex-related differences were not significant.

Older adults who regularly engaged in exercise or leisure time physical activities of at least moderate intensity were excluded during recruitment for the study. It may therefore be assumed that phases of higher activity throughout the day were mostly due to basic (e.g. bathing, dressing, eating) and instrumental activities of daily living (e.g. housework, shopping), or social activities. Such activities constitute an integral part of the daily routine. Studies showed that daily routines may improve health and well-being [32, 33], and are strongly associated with sleep quality in older adults [34]. We hypothesize that the regular integration of exercise or leisure time activity on a daily basis, i.e., the establishment of an “exercise routine”, would also contribute to enhanced sleep quality, health and health-related quality of life in older adults with sedentary or low activity levels.

In order to create routines, it would seem to be necessary to identify spare time for leisure time physical activity or exercise that does not compete with existing important routines and commitments in the course of the day. The present study revealed a slight peak between 3 and 4 pm, and a sharp decrease in physical activity from 4 pm on. One option might be prolonging this relatively short phase of higher physical activity in the afternoon by implementing physical activity programs during this time of the day. Since the explorative analysis of diurnal physical activity behavior revealed relatively high inter-individual variation (reflected by the wide confidence intervals [see Figures 2, 3, 4, 5, 6 and 7]), individualized approaches might be another, probably even more promising option to integrate an optimized physical activity or exercise routine into older adults’ lives.

### Diurnal physical activity profiles for subgroups

Sex, age, morbidity, and season had no moderating effect on diurnal physical activity profiles of older adults. A mobility limitation was the only factor with both, a significant effect on overall physical activity levels and on diurnal activity profiles, irrespective of participants’ sex, age, and morbidity, and the season. Gait unsteadiness due to a loss of lower-body strength and balance, and resulting fear may be an explanation for the overall decreased activity in older people who depend on a walking aid. Several studies showed that using walking devices is independently associated with being more fearful of falling [35, 36]. Fear of falling has in turn been reported as a barrier to physical activity [13, 37, 38]. In those with moderate or severe mobility limitation, fear of falling has been even more frequently reported as a barrier to exercise [14]. It has been shown that fear-induced activity reduction [39] or the need for a walking aid [40], and the tendency to adopt a more sedentary lifestyle accelerate functional deconditioning and disability.

The adjusted profile for older adults using a walking aid suggests that their lower overall physical activity may be partly attributable to the lack of a second activity peak in the afternoon. This activity plateau might be one starting point for increasing physical activity levels in older adults using walking aids. Balancing possibilities for accessing inactive older adults with functional limitations, Schofield et al. [41] report that the general practitioner (GP) is the most trusted source of physical activity information, especially for this target group. A GP is one of the few persons who has regular access to mobility-limited older adults and often has trusting long-lasting relationships with those patients [42]. The GP may play a central role in promoting physical activity to this hard-to-reach target group, namely hardly active or sedentary, chronically ill and mobility-limited older adults. The GP could raise their awareness of the necessity of physical activity and opportunities to become more active, what could help them increase their physical activity and manage their health.

### Strengths and weaknesses

The present study targeted community-dwelling, though chronically ill and mobility-limited older adults with a low physical activity level. They are difficult to reach for exercise interventions, as many of them rarely leave their homes [43]. The success of the approach to access and recruit this hard-to-reach target group for an exercise intervention via their GP was unclear. A selection towards fitter and healthier older adults in intervention studies is a known research problem [44]. In our study, all older adults over the age of 70 years who visited their GP within the past 6 months, and fulfilled the predefined inclusion criteria were given the chance to participate in our study. Nonetheless, a certain selection towards healthier and fitter older adults during the multi-stage recruitment for the trial, e.g. the phase of response to the invitations for final eligibility screening, cannot be excluded. Additionally, participants dropped out from our study, if they did not fulfil certain analysis criteria (see methods section). The flow chart, however, indicated that sex and age did only marginally change from the 245 eligible participants to the 149 patients analyzed. We aimed to reach as high representativeness as possible regarding the recruitment of a sample of chronically ill and mobility-limited older adults with low physical activity levels. Our results should be regarded as relevant for a population of older primary health care patients who fulfil eligibility criteria for the present study, and who would participate in an exercise intervention study in their GP’s practice.

The present study assessed physical activity by means of the pedometer OMRON Walking Style Pro. Exact pedometer wear time was not documented by participants in order to not overstrain them with documentation demands. Instead of that, participant documented whether they wore the device for the whole day. The lack of a definite start and end point of physical activity lowers the validity of data on calculated average steps per hour. This is a limitation of the study. The accuracy of the specific pedometer model has already been demonstrated in young adults [45] and middle-aged adults [24]. Some studies, however, have reported reduced accuracy for slower walking [46–48]. This could affect physical activity measurement in the present sample of mobility-limited older adults. We tried to maximize precision by using a piezoelectric pedometer. These sensors were shown to be more accurate in slow walking speeds than spring-levered pedometers [49]. They are thus more appropriate for use in elderly and special populations. Additionally, participants were instructed to wear the device at the waistband/belt which was shown to be the most precise of the four proposed mounting positions [24]. Anyway, an underestimate of actual steps cannot be excluded. However, it may be assumed that while a measurement error of this nature would affect the absolute level of physical activity, it would not affect the distribution throughout the day. While the total physical activity levels reported in this study should therefore be interpreted with caution, the diurnal profiles of physical activity may be regarded as a true reflection of older adults’ daily physical activity rhythm.

On the other hand, the objective pedometer-based physical activity measurement is an advantage of the present study. Pedometer measurement is not subject to recall or social-desirability bias [50]. Additionally, pedometers are considered to be very appropriate for use in older adults or patients because they are very easy to handle [51]. The lack of qualitative information on the content of physical activity throughout the day, however, is a limitation of the present study. Further studies on older adults’ diurnal physical activity profiles should include log entries, for instance, in order to better understand objectively measured physical activity behavior over the course of the day.

Despite these limitations, this is the first study to explore diurnal profiles of physical activity in community-dwelling, chronically ill and mobility-limited older people, which allows conclusions on optimized physical activity-promotion in this target group to be made.

## Conclusions

The present findings allow suggestions to be made to optimize scheduling of leisure time physical activities and exercise in accordance with older adults’ daily rhythm. Prolonging periods of elevated physical activity in the afternoon while respecting individual daily routine and commitments could be an option for facilitating the integration of physical activity and for making it a habit in older adults’ daily lives. The use of a walking aid seems to be an indicator for a quite low activity plateau during the second half of the day. People who use walking aids should be motivated to increase their physical activity during afternoon; this might help to increase the overall low physical activity level of this vulnerable subgroup of older adults.
